# Intravenous pyogenic granuloma in the internal jugular vein: A case report and literature review

**DOI:** 10.1097/MD.0000000000024570

**Published:** 2021-02-12

**Authors:** Yuheng Yang, Xiaoping Ye, Binjie Fu, Zhui Li, Yangyang Feng, Yu Zhao, Hong Liu

**Affiliations:** a Department of Vascular Surgery; b Department of Ultrasound; c Department of Radiology, The First Affiliated Hospital of Chongqing Medical University, Chongqing, China.

**Keywords:** imaging examination, internal jugular vein, intravenous pyogenic granuloma

## Abstract

**Rationale:**

Intravenous pyogenic granuloma (IVPG) is a special type of pyogenic granuloma, and its preoperative diagnosis is difficult. We report a rare case of IVPG that develops in the lumen of the internal jugular vein (IJV). Here, we analyze the imaging characteristics of present case and summarize the imaging characteristics of previous reported cases.

**Patient concerns:**

A 44-year-old man who presented with a growth in the IJV without any symptoms.

**Diagnoses:**

A diagnosis of IVPG was made, based on the pathological examination after surgery.

**Interventions:**

The patient underwent surgery to excise the vein segment containing the neoplasm.

**Outcomes:**

The patient did not present with any complications in the postoperative follow-up period.

**Lessons:**

For clinician, IVPG's preoperative diagnosis is difficult. Although histopathology remains the gold standard for diagnosis, the combination of multiple types of imaging examinations is necessary to rule out the differential diagnoses of IVPG.

## 1 Introduction

Lobular capillary hemangioma (LCH) [pyogenic granuloma (PG) or granulation tissue-type hemangiomas] is a benign tumor that develops commonly on the skin or mucous membrane and is red or violaceous in color and shows polypoid growth. It is neither caused by infection nor a real inflammatory granuloma. Intravenous pyogenic granuloma (IVPG) is a special type of PG that usually develops in the veins of the neck and upper extremities, such as the jugular vein,^[1–3]^ cephalic vein,^[4]^ azygos vein,^[5]^ and some special areas like the renal vein.^[6]^ IVPG arising from the internal jugular vein (IJV) is quite rare. We report a rare case of IVPG arising from the IJV in our center.

## 2 Case report

A 44-year-old man had a nodule on the left IJV without any symptoms based on a routine examination by ultrasonography. There were no abnormalities on medical history and physical examination. In addition, laboratory test results were normal.

**Figure 1:**
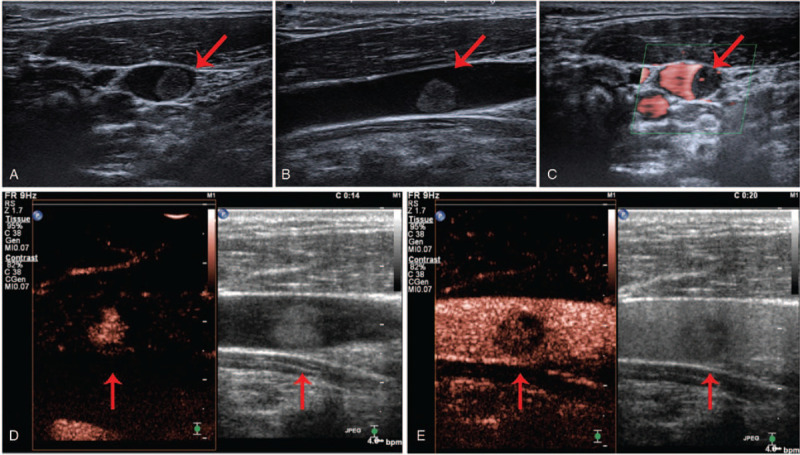
Ultrasonography: The image of the 2-dimensional ultrasound transverse section (A) and longitudinal section (B); (C) The image of the superb microvascular imaging; The images of the arterial phase (D) and the venous phase (E) of contrast-enhanced ultrasonography. The red arrows are pointing to the neoplasm.

According to the doppler ultrasound examination, an abnormal echo (mainly with hypoecho) was probed in the middle section of the left IJV, with a clear boundary and slightly irregular shape. Its dimensions were 8.7 × 7.7 × 6.7 mm, connecting to the posterior lateral wall of the IJV, and swinging with the pulse of the vessel (Fig. 1A, B). Color Doppler flow imaging showed color filling defect within the IJV, and superb microvascular imaging detected star-like flow signal within the nodule (Fig. 1C). Contrast-enhanced ultrasonography showed contrast filling defect in the lesion. The abnormal echo was unevenly enhanced during the arterial phase, and the time of enhancement was later than in the internal carotid artery and earlier than in the IJV. Enhancement was observed initially in the junction of the neoformation and posterior lateral wall of the IJV and then diffusely toward the free surface, without enhancement in some areas inside the nodule, and uneven low enhancement was shown during the venous phase (Fig. 1D, E). To further identify the characteristic of the neoformation, computed tomography (CT) and magnetic resonance imaging (MRI) were performed.

**Figure 2:**
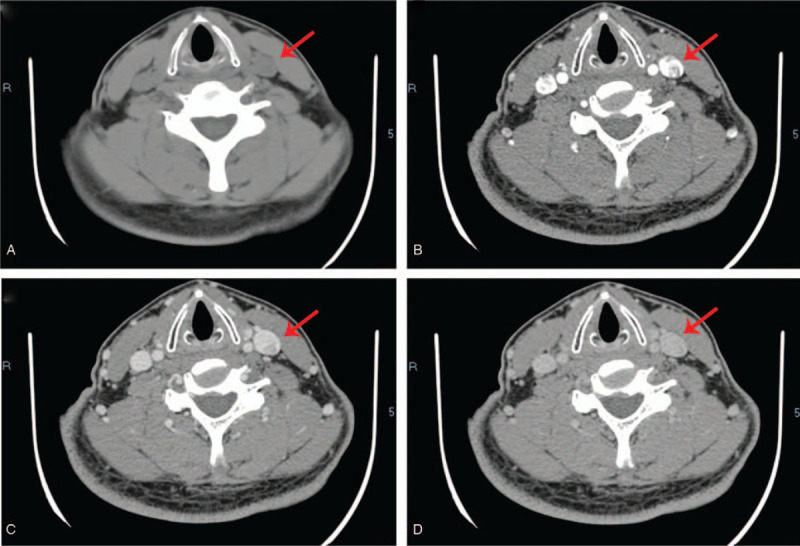
Enhanced CT of neck: (A) CT plain scan; (B) The image of enhanced CT arterial phase; (C) Venous phase; (D) Delayed phase. The red arrows mark the neoplasm.

CT demonstrated that there was an isodense nodule in the proximal lumen of the left IJV, the size of which was approximately 8.5 × 5.9 mm, with a CT value of 40 HU. Thin-layer CT showed increased tortuosity and small vessels shadowing within the nodule during arterial phase enhancement (Fig. 2B) with a CT value of 92 HU. In addition, continuous “filling” enhancement was observed during the venous and delayed phases, with the enhancement degree consistent with the venous density, and the CT value was 109 HU. There were no enlarged lymph nodes in other parts of the neck (Fig. 2A--D).

**Figure 3:**
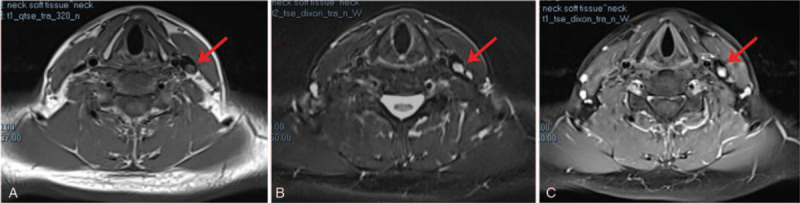
Contrast-enhanced magnetic resonance imaging (MRI) images: (A) The neoplasm is isointense on T1-weighted imaging; (B) The neoplasm is hyperintense on T2-weighted imaging; (C) Enhanced MRI image on T1-weighted imaging. The red arrows mark the neoplasm.

On MRI, a pedunculated nodule was observed protruding into the lumen at the initial segment of the IJV, with clear boundary and smooth edge without obvious invasion to the adjacent venous wall. Subsequently, it was significantly enhanced, and its dimension was approximately 8.2 × 5.9 mm. The nodule was isointense on T1-weighted imaging but hyperintense on T2-weighted imaging (Fig. 3A--C).

**Figure 4:**
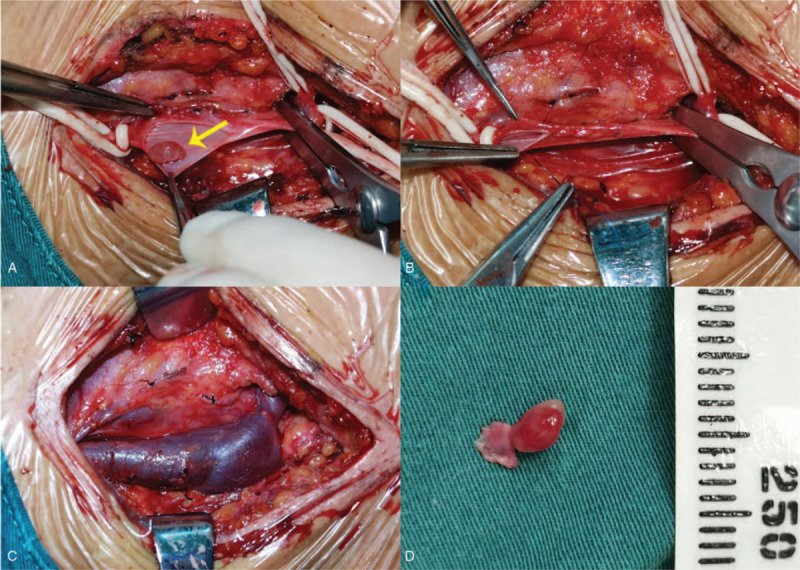
Intraoperative observation: (A) Surgical field showing the neoplasm in the internal jugular vein (yellow arrow); (B), (C) The reconstruction of internal jugular vein; (D) Excised IVPG-containing internal jugular vein segment.

Therefore, the possibility of intracavitary thrombosis could be ruled out, and we suggested that the neoformation was a primitive endoluminal neoplasia. Subsequently, the patient underwent surgery with general anesthesia to excise the neoformation and explore the area around the left IJV. We found a pedunculated red neoformation with soft texture, which measured approximately 5 × 3 mm within the left IJV after cutting open the left IJV (Fig. 4A). A reddish, enlarged lymph node with soft texture was also found beside the left IJV. Finally, the neoformation was excised with the coterminous vein segments (Fig. 4D), and the lymph node was also excised. The left IJV was reconstructed subsequently and revealed good patency after being unblocked (Fig. 4B, C). The patient recovered well postoperatively without any complications.

**Figure 5:**
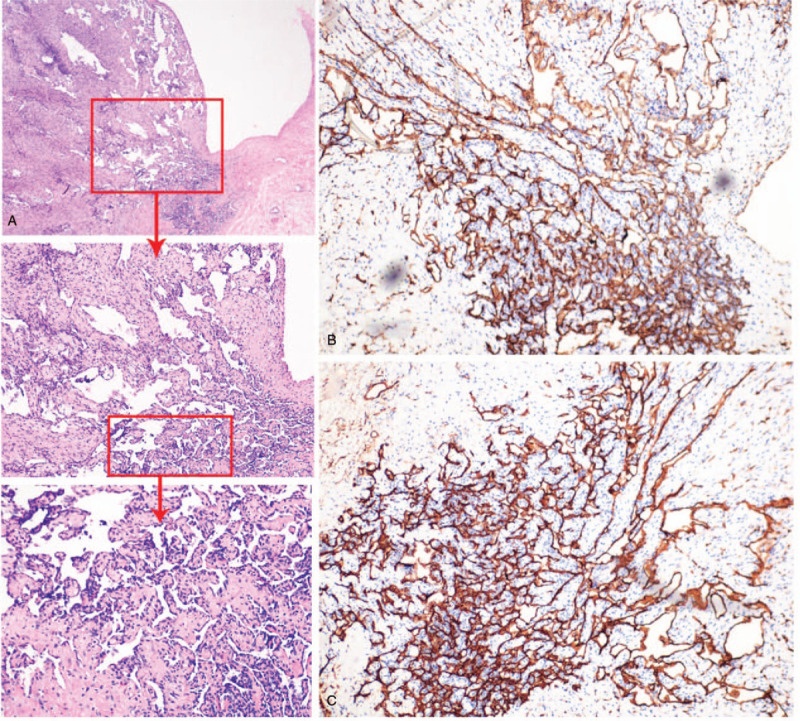
(a) Histology slides of the neoplasm at 10 × , 20 × , 40 × magnification, respectively (Hematoxylin-eosin staining [H&E]); (B), (C) CD31, CD34 stain showing distribution of vessels in neoformation (20 × magnification).

Histological examination results showed a large number of proliferating capillaries, presenting with lobular distribution. Endothelial cells of the capillaries were flat, surrounded by fibrous tissue and myxoid stroma. Moreover, there was a small amount of chronic inflammatory cell infiltration. Immunohistochemical staining results showed CD34 (+), CD31 (+), ERG (+), VIM (+), KI67 5% (+), and SMA (−) (Fig. 5A--C). The lymph node that was excised from the neck was diagnosed as reactive hyperplasia. The patient did not present with any complications during the 3-month follow-up period.

## 3 Discussion

In 1982, Mulliken and Glowacki classified the previous definition of “haemangioma” into vascular tumors and vascular malformations based on whether there was abnormal proliferation of endothelial cells.^[7]^ LCH is one of the benign vascular tumors that frequently develop in the skin and mucous membranes. Most scholars supposed that LCH's development may be related to trauma, drugs, hormone changes, and production of vascular endothelial growth factor, but there is no clear evidence to prove their exact relationship. IVPG, as a special type of LCH, mainly develops in children and young adults.^[1]^ Most patients are asymptomatic and have no obvious inducement, usually discovered during routine health checkup or investigations focused on other pathologies. In our literature review, we have found 5 cases of vascular lesions arising from the IJV and provided relevant summaries (Table 1).

**Table 1:** Summary of the cases of vascular lesions arising from the IJV reported (last checked August 2020).

References	Age, yr/Sex	Medical history	Imaging performance	Histopathology	Diagnosis
Duggal et al ^[10]^	27/male	a gradually enlarging mass in left supraclavicular area for the last 3 years; no associated symptoms	DUS: the left common carotid artery was displaced posteriorly by a supraclavicular mass; the mass closely abutted the artery; no luminal narrowing of carotid artery. MRI: a heterogenous well-defined mass, 7 × 4 × 6 cm in size in infra hyoid neck on the left side; the lesion appeared isointense to hypointense to muscle on T1-WI and hyperintense on T2-WI and STIR images with fluid-fluid level; the left IJV superior to the mass was well defined and at the level of the mass was compressed; there was hyperintense signal in the left IJV secondary to flow related phenomenon.	many dilated, ectatic vascular channels with muscular walls; the intervening fibroadipose tissue showed scattered chronic inflammatory cells and focal calcification.	vascular malformation
Wu et al ^[1]^	38/female	a nodule within the right IJV by an ultrasonography for 15 days	CT: a high-density nodule on the medial and posterior wall of the right IJV, the diameter of which was about 5.7 mm. DUS: a well-defined nodular mass with abundant blood supply that involved the IJV.	a thin layer of smooth muscle cells under the endothelial cells of the neoplasm: in particular, a lobular proliferation of capillaries could be found; CD34(+); smooth muscle-specific actin(+)	intravenous pyogenic granuloma
Al-Natour M et al ^[8]^	87/male	discomfort in the left neck; dyslipidemia; hypertension	DUS: a homogenous hypoechoic mass within the lumen of the left IJV. CT: a mass described as a possible lymph node in the neck or a neoplasm.	a cavernous hemangioma of the IJV wall	cavernous hemangioma
Cera C et al ^[2]^	51/male	a neoformation was accidentally discovered inside the left IJV; gynecomasty; mastectomy; hypothyroidism	DUS: a markedly hypoechoic, vascularized, and apparently pedunculated neoformation. CT: an IJV nonocclusive intralumen neoformation with an oval, moderately irregular, and uneven morphology; the lesion was stuck on the posterioremedial face of the mentioned vein, and its dimensions were about 10 × 8.7 × 7.6 mm.	the neoformation was made up by myxoid stroma and little vascular structures, similar to capillaries;CD31(+);CD34(+)	pyogenic capillary hemangioma
Li et al ^[3]^	55/female	a nodule in the left side of her neck for 3 mo	CT: a filling defect in the left IJV	vascular expansion, and significant proliferation of the vascular endothelium; a large number of lymphocytes, plasma cells, and neutrophils were present along with a lymphoid follicle in the process of formation; CD31(+);CD34(+);CD68 (tissue cells +);Ki−67(germinal center+ >90% and the germinal center <5%);F8(+)oven; CK(-).	granulation tissue–type hemangioma

CT = computed tomography, DUS = doppler ultrasound, IJV = internal jugular vein, MRI = magnetic resonance imaging.

According to the literature, there were 3 cases ^[1–3]^ confirmed as IVPG based on the final histopathological examination. Four patients, who had vascular lesions originating from the IJV, had no symptoms, except 1 patient with discomfort in the left neck.^[8]^ In addition, only 1 patient had a previous history of hypothyroidism, whose PG within the IJV was considered to be the result of related hormonal disorder.^[2]^ All the 5 patients’ results of the laboratory tests were basically normal.

Currently, there are no special requirements for preoperative diagnostic examination due to the rarity of the vascular lesions arising from the IJV. However, ultrasonography seems to be the preferred method for a first-level differential diagnosis because it is noninvasive and convenient. In all cases, ultrasonography showed a hypoechoic nodular mass with abundant blood supply, which is consistent with the present case. Moreover, the contrast-enhanced ultrasound showed that the nodule was unevenly enhanced during the arterial phase in this case, and the enhancement time was later than in the internal carotid artery and earlier than in the IJV. We suspected that this may be related to the fact that IVPG is composed of proliferated capillaries, leading to a certain specificity in the strengthening time, which may be the basis for differentiation from other lesions. As there are few related cases and the previous literatures are insufficiently detailed in reporting manifestation of CT, it is difficult to summarize the features of IVPG (arising from the IJV) in CT. Tortuous small vascular shadows in the nodule were observed during the arterial phase of CT in the present case, which also suggests that the tumor is mainly composed of neonatal blood vessels. On MRI, the nodule was isointense on T1-weighted imaging but hyperintense on T2-weighted imaging. The nodule tends to be benign on a combination of imaging findings, and differential diagnoses included venous thrombosis, intravascular papillary endothelial hyperplasia (IPEH), or any other vascular tumor. IPEH is mainly characterized by sheet-like hyperintensity on T1-weighted imaging and polycystic nodule-like hypointensity on T2-weighted imaging,^[9]^ so it can be distinguished from the present case. Thus, it is undeniable that imaging examinations play an indispensable role in preoperative differential diagnosis and can assist in qualitative diagnosis.

Surgical excision is the main treatment for IVPG arising from the IJV, which involves complete excision of the vein segment containing the neoplasm.^[8]^ In the present case, we reconstructed the IJV after excising the neoplasm and the connected vein wall due to small neoplasm and localized coloboma of the IJV. Nevertheless, whether to reconstruct the vein mainly depends on the site and degree of neoplasm involvement. In some veins, such as the vena cava, venous reconstruction may be required to restore venous continuity.^[2]^

Primary IVPG arising from the IJV is a rare subtype of vascular tumor without characteristic epidemiological and endocrinological features. Although histopathology remains the gold standard for diagnosis, preoperative imaging examination is necessary, and the combination of multiple types of imaging examinations is conducive to the differential diagnosis of IVPG. In the future, the detection rate of IVPG may increase with the improvement of imaging technique and people's health awareness. Therefore, it is indispensable to summarize the imaging characteristics of IVPG developing in various parts of the body.

## Author contributions

**Data curation:** Binjie Fu, Zhui Li, Yangyang Feng, Yu Zhao, Hong Liu.

**Investigation:** Yuheng Yang, Hong Liu.

**Methodology:** Yuheng Yang, Xiaoping Ye, Hong Liu.

**Project administration:** Yuheng Yang.

**Resources:** Binjie Fu, Zhui Li, Yangyang Feng, Yu Zhao, Hong Liu.

**Software:** Xiaoping Ye.

**Supervision:** Hong Liu.

**Validation:** Xiaoping Ye.

**Visualization:** Xiaoping Ye.

**Writing – original draft:** Yuheng Yang.

**Writing – review & editing:** Yuheng Yang, Xiaoping Ye.

## Abbreviations

Abbreviations: CT = computed tomography, IJV = internal jugular vein, IPEH = intravascular papillary endothelial hyperplasia, IVPG = intravenous pyogenic granuloma, LCH = lobular capillary hemangioma, MRI = magnetic resonance imaging, PG = pyogenic granuloma.
